# The Role of Bronchoscopy in the Management of Lung Nodules. Is it too Early to Retire Transthoracic Needle Aspiration?

**DOI:** 10.2174/0118743064371372250709073630

**Published:** 2025-07-14

**Authors:** Munish Sharma, Swikriti Shrestha, Yasoda Rijal, Salim Surani

**Affiliations:** 1 Division of Pulmonary, Critical Care and Sleep Medicine, Baylor Scott and White, Texas, USA; 2 Internal Medicine, Universal College of Medical Sciences and Teaching Hospital, Bhairahawa NPL; 3 Internal Medicine, Tribhuvan University Teaching Hospital, Kathmandu, NPL; 4 Division of Pulmonary, Critical Care and Sleep Medicine, Adjunct Clinical Professor, Texas A&M University, USA

**Keywords:** Lung nodule, Transthoracic needle aspiration, Endobronchial ultrasound biopsy, Robotic bronchoscopy, Lung carcinoma, Virtual bronchoscopic navigation

## Abstract

Transthoracic Needle Aspiration (TTNA) is a medical procedure that involves the percutaneous insertion of a needle, typically guided by computed tomography, to aspirate lung tissue. During TTNA, the needle is often directed through the pleura and lung to reach and aspirate target tissues, such as the lung or lymph nodes. This procedure is commonly used to obtain tissue samples for diagnostic purposes. Over the past decade, invasive bronchoscopy, including endobronchial ultrasound biopsy (EBUS), radial probe, navigational, and robotic bronchoscopy, has improved the yield and safety profile of lung nodule biopsies while ensuring adequate lung tissue sampling. In this review article, we aim to compare the differences in efficacy, safety, and diagnostic yield among various diagnostic modalities in pulmonary medicine.

## 1 INTRODUCTION

Lung cancer is the second most common form of cancer and the leading cause of cancer-related deaths [1]. A landmark study in 2011, known as the National Lung Screening Trial (NLST), demonstrated a 20% reduction in lung cancer-specific mortality using Computed Tomography (CT) scanning to screen at-risk populations [2]. As a result of this study, screening at-risk individuals using CT scanning has become the standard of care, leading to an increase in the identification of incidental lung nodules that require further evaluation and workup. TTNA has been a significantly crucial diagnostic modality for various pulmonary pathologies. TTNA was initially documented over 100 years ago by Leyden [3] to detect an inflammatory process. In 1886, Menetrier utilized this method to identify a pulmonary neoplasm. The use of fluoroscopically guided TTNA for diagnosing lung cancer gained prominence in the 1960s because of Dahlgren and Nordenstrom [3]. Haaga and Alfidi [4] were the first to outline a CT-guided lung biopsy in 1976. Over the years, various imaging modalities have been used to guide the needle to the location of aspiration or biopsy, including plain radiograph, fluoroscopy, computed tomography, ultrasound-guided [6, 7], and CT-guided electromagnetic navigational TTNA (E-TTNA). In addition to these modalities, various image-guided bronchoscopic techniques have also emerged to improve diagnostic yield and reduce complication rates compared to conventional bronchoscopic biopsies. These include robotic bronchoscopy (RB), Virtual Bronchoscopic Navigation (VBN), Electromagnetic Navigation Bronchoscopy (ENB), Radial Probe Endobronchial Ultrasound (RP-EBUS), and ultrathin bronchoscopy [7].

## 2 METHODOLOGY

This review is based on a comparative analysis of CT-TTNA and bronchoscopic modalities for pulmonary nodule diagnosis. We performed a comprehensive review of peer-reviewed articles, clinical guidelines, and recent studies to evaluate the diagnostic accuracy, clinical utility, and safety of CT-TTNA and bronchoscopic techniques, including EBUS, RB, and ENB. The modalities were compared in terms of key parameters, such as sensitivity and overall accuracy for peripheral and central lung lesions. The safety profile for modalities and accessibility in clinical practice was also compared.

### 2.1 Core Tips

Computed tomography-guided transthoracic needle aspiration (CT-TTNA) is used to diagnose suspicious lung lesions. Lung nodules are detected by early screening, and the treatment for lung carcinoma is highly personalized based on genetic markers in the sample for non-small cell lung cancer. This can be challenging to obtain through TTNA alone due to the smaller sample size, which may not be enough to run the specimens for the tumor markers. Even with adequate samples, we must follow up with the advanced bronchoscopy method for staging. With the availability of advanced fluoroscopic bronchoscopy and the complications related to TTNA, performing TTNA has become marginalized. Considering the lower diagnostic yield, lower Negative Predictive Value (NPV), higher contraindications, and higher complications, it may be time to choose other advanced diagnostic modalities over CT-guided TTNA.

### 2.2 Diagnostic Yield and Safety of Various Diagnostic Modalities in Lung NodulesCT-guided TTNA/TTNB

A meta-analysis of 48 studies by DiBardino *et al.*, encompassing 10,383 cases, found that Transthoracic Needle Aspiration (TTNA) demonstrated a sensitivity of 88.7% for lesions smaller than 1 cm, especially pleural-based lesions. Although TTNA showed a specificity of 100% for lung cancer, its negative predictive value (NPV) was only 77% at best. Therefore, if TTNA results are negative, further investigations must confirm the diagnosis [8]. In a study involving 687 patients, the diagnostic yield of TTNA for Ground Glass Opacities (GGO) ranged from 80% to 97% [8]. Despite this, the 2017 Fleischner Society Guidelines do not recommend TTNA for GGO due to concerns about inadequate sampling and false-negative results.

In a retrospective study conducted by Zhang *et al.* on 513 patients, the diagnostic efficacy and complication rates of CT-TTNA were compared with those of rEBUS-TBB. CT-TTNA showed higher rates in the positive diagnostic aspect (*P* = 0.001). In rEBUS-TBB, larger lesions (>2 cm) had a higher positive diagnostic rate (*P*=0.012) while lesions closer to the chest wall had lower rates (*P*=0.046). For CT-TTNA, lesion size did not significantly affect the diagnostic rate; however, different segments showed significant differences in rates (*P* = 0.044). Pneumothorax incidence was lower for lesions near the chest wall in CT-TTNA (*P*=0.037). The overall success rate for rEBUS-TBB was 87.4%, with better outcomes for larger lesions and those with bronchial signs. CT-TTNA is preferred for smaller lesions near the chest wall, whereas rEBUS-TBB is better for larger, distal lesions or those with bronchial signs [9, 10].

A retrospective study conducted by Chaudry *et al.* involving 1006 patients revealed that the diagnostic effectiveness of TTNA surpassed that of ENB, particularly in identifying pulmonary nodules. For lesions larger than 2 cm, the sensitivity of CT-TTNA was 87.4%, with a specificity of 46.2%, a positive predictive value of 94.7%, and a negative predictive value of 25.0%. In contrast, the sensitivity of ENB was 56.3%, specificity 100.0%, positive predictive value 100.0%, negative predictive value 41.7%, and overall accuracy was 66.7%, with a p-value of 0.001. However, the rate of occurrence of pneumothorax was higher in the CT-TTNA group (15.6%) compared to ENB cases (3.85%), showing a statistically significant difference (p = 0.001) [11].

In a retrospective cohort study conducted by Munoz *et al.*, involving 830 patients, EBUS was found to be the preferred initial method for diagnosing lung cancer. When assessing the need for additional biopsies and the rate of complications compared to EBUS, TTNA exhibited a higher percentage in both areas, with an adjusted odds ratio (OR) of 8.63, a 95% confidence interval (CI) of 3.12-23.8, and a p-value of <0.001 for the necessity of a second biopsy, as well as an adjusted OR of 128.21, a 95% CI of 20.2-812.4, and a p-value of <0.001 for the complication rate. However, since EBUS is not available at all healthcare facilities due to either insufficient resources or a lack of trained interventional pulmonologists, following guideline-recommended procedures can be challenging in every situation. In such instances, TTNA remains a viable diagnostic option [12].

Currently, diagnosing lung carcinoma requires understanding molecular changes and testing for mutations like EGFR, BRAF, and KRAS G12C, as well as rearrangements in various genes. Treatment has advanced with multiple generations of tyrosine kinase inhibitors tailored to these mutations. Achieving this requires sufficient sampling for Rapid Onsite Evaluation (ROSE), Hematoxylin and Eosin (H&E) staining, Papanicolaou staining, Fluorescence In Situ Hybridization (FISH), immunohistochemistry, and Polymerase Chain Reaction (PCR) testing, which TTNA cannot provide [13, 14].

According to a study by Vanderlaan *et al.* in 2014, when the tissue samples were obtained from image-guided transthoracic core-needle biopsies, the failure rate to detect tumor genotype was substantially higher than other techniques [15]. Almost 20% of patients undergoing TTNA experienced pneumothorax, with 50% of those needing chest tube insertion. Other complications included hemorrhage, air embolism, and pulmonary bleeding occurring in about 11% of cases [9, 16]. Limitations of the procedure include the need for bronchoscopy after CT-TTNA and issues with diagnosing specific nodules. Pneumothorax risk factors, include emphysema, longer needle paths, lower lobe location, increased passes for additional biopsy, and small lesions (<2cm). Using real-time guiding modalities, like CT fluoroscopy or Cone Beam CT (CBCT), can help reduce pneumothorax rates, particularly in patients with severe emphysema, by decreasing the number of pleural punctures and improving procedural accuracy [17-19]. A 2022 study in Frontiers in Medicine found that real-time tracking of needles during biopsies reduces the risk of incorrect placement, lowers complications, and improves the chances of obtaining a diagnostic sample. It also emphasized that using thin-slice CT imaging (up to 2.5 mm thickness) enhances image resolution for better visualization and precise needle placement. Additionally, combining thin-slice imaging with advanced reconstruction methods increases the ability to identify small lesions, improving diagnostic yield and reducing the need for repeat procedures [20].

The ongoing VERITAS trial (Navigation Endoscopy to Reach Indeterminate Lung Nodules versus TTNA) is a randomized controlled trial to compare ENB with CT-TTNB for the diagnosis of peripheral lung nodules measuring 10-30 mm in diameter, with a pre-test probability of malignancy of at least 10%. It aims to provide valuable data on the diagnostic approach to lung nodules, which frequently represent the earliest and most treatable stage of lung cancer [21].

Other factors that influence the diagnostic yield of CT-TTNB are listed below:

1. **Size of lesion**: The size of pulmonary nodules significantly affects the diagnostic yield in CT-TTNB. Larger lesions (41–50 mm) exhibit a high diagnostic accuracy of 95%, although accuracy drops to 80% for those exceeding 50 mm, possibly due to central necrosis and complications arising from surrounding lung tissue [22-24]. Smaller lesions, particularly those under 10 mm, lead to reduced diagnostic yields, with a success rate of 60% for nodules measuring 15 mm or less, compared to 82% for those larger than 15 mm. This decrease in accuracy is mainly due to difficulties in precise targeting during biopsy procedures [22-26].
2. **Location of the nodule:** A study by Yeow *et al.* found that the depth of lung lesions is a key predictor of pneumothorax (PNX), with lesions contacting the pleural surface having the lowest risk of PNX. In contrast, subpleural lesions between 1 mm and 20 mm deep demonstrated the highest risk, which decreased as depth increased. Recommendations include using longer, oblique needle paths to minimize risks [27]. Khan *et al.* also noted a higher PNX rate for lesions within the lung parenchyma compared to those in the pleura, emphasizing the importance of lesion proximity in predicting severe outcomes [28]. Additionally, fine-needle aspiration techniques indicate that traversing more lung parenchyma during biopsy increases complication risks, underscoring the need for careful management in CT-TTNB procedures [29, 30].
3. **Needle path:** The length of the needle path is crucial in biopsy procedures, impacting both precision and complications. Shorter needle paths, found in lesions closer to the pleural surface, enhance diagnostic accuracy by reducing the distance the needle travels and minimizing deviation. A 2022 study reported that shorter paths are associated with better diagnostic yield and fewer nondiagnostic outcomes, especially for smaller nodules. Conversely, longer needle paths, required for deeper lesions, increase the likelihood of complications and can affect the biopsy's overall effectiveness [30-32].
4. Several factors significantly influence the decision-making process for selecting patients to undergo CT-guided TTNA/ TTNB. Key considerations include the patient's financial situation, which can determine their access to these procedures and related care. Additionally, the availability of alternative diagnostic modalities, such as MRI or PET scans, may impact whether a CT-based approach is deemed necessary or appropriate. Lastly, the expertise of the institution and the medical professionals performing the procedures plays a crucial role in ensuring that the right patients are chosen for these interventions, thereby affecting the overall diagnostic yield of CT-TTNA/TTNB. Collectively, these factors contribute to optimizing patient outcomes and maximizing the effectiveness of the diagnostic process [33, 34].

### 2.3 Virtual Bronchoscopic Navigation (VBN)/ Electromagnetic Navigation Bronchoscopy (ENB)

According to a pooled study of 13 research works, procedures using VBN assistance for biopsy of peripheral pulmonary lesions had an overall diagnostic yield of 74%, and lesions less than 2 cm had a reduced yield of 67%. According to a meta-analysis of 39 investigations where VBN was combined with other image-guided techniques, a diagnostic yield of 72% was found [35-37]. According to a prospective study by Folch *et al.*, across 29 centres, 1215 patients underwent an ENB procedure, and the overall diagnostic yield of ENB was 72.9%. The pneumothorax rate was 4.9%, with 2.9% requiring chest tube placement. The diagnostic yield was higher for larger lesions and those located in the upper lobes of the lungs [35].

### 2.4 Cone-Beam Computed Tomography Bronchoscopy

A retrospective study by Patel *et al*. compared 240 nodules in the CBCT group and 233 in the TTNA group. Patient and target nodule characteristics were similar across groups. Furthermore, the overall diagnostic accuracy was not significantly different (87.1% for CBCT *vs*. 84.9% for TTNA, p=0.510). However, the CBCT group had significantly fewer pneumothoraces requiring intervention (0.4% *vs*. 8.6%, *P* < 0.001) and fewer pneumothoraces managed by observation (1.3% *vs*. 30.5%, *P* < 0.001). Procedures with CBCT took longer (median, 69 min *vs*. 32 min, *P*<0.001) due to EBUS-guided staging in 63.3% of cases. Diagnostic follow-ups were needed for 7 patients in the CBCT group and 12 in the TTNA group. Although there appear to be no significant differences in diagnostic outcomes, further research is necessary to clarify any possible differences between the two biopsy methods and to gain a better understanding of the situations in which each technique might be preferred [36].

### 2.5 Endobronchial Ultrasound (EBUS)

A prospective study by Zhu *et al.* on 327 cases comparing the diagnostic yield of CT-TTNA versus Endobronchial Ultrasonography with a Guide Sheath (EBUS-GS) for the diagnosis of peripheral lung lesions revealed that both procedures had limitations. EBUS-GS had a lower diagnostic rate but fewer complications, while patients tolerated CT-TTNA better. Based on lesion size, the recommendation is to use EBUS-GS for lesions with a diameter of 30 mm or less and CT-TTNA for lesions larger than 30 mm. Additionally, CT-TTNA samples were found to be more advantageous for genetic testing [37]. In a study by Asahina *et al.*, which included 30 peripheral pulmonary lesions with diameters ranging from 2 cm to 3 cm, virtual bronchoscopy combined with radial EBUS-GS yielded a diagnostic yield of more than 90%, comparable to CT-TTNB [38]. A study by Ho *et al.* reported that rEBUS-TBB (radial endobronchial ultrasound-guided transbronchial biopsy) is significantly safer, with a five to eight times lower risk of complications, including pneumothorax, hospital admission, bleeding, and composite complications [39].

### 2.6 Robotic Bronchoscopy

A study involving 131 cases found that shape-sensitive Robotic Bronchoscopy (ss-RAB) has a sensitivity of nearly 80%, increasing for lesions larger than 1.8 cm and those located centrally [40]. A meta-analysis indicated a pooled diagnostic yield of 85.2% with minimal complications, suggesting its use in the biopsy of pulmonary nodules [41]. In another study involving 52 patients and 59 lesions, the combination of robotic bronchoscopy with Cone Beam CT (CBCT) achieved an 83% diagnostic yield and 86% sensitivity, particularly effective for peripheral lesions [42-44]. Three large meta-analyses reported diagnostic yields ranging from 80.4% to 84.3%, with more extensive lesions (>20 mm) showing higher yields. Pneumothorax occurred in 2.3% of cases, with 1.2% requiring chest tubes. The findings indicated that newer modalities are safe and effective for diagnosing pulmonary lesions, especially when nodule size and bronchus sign are favorable [45-47].

Despite its known advantages, current research findings indicate that the diagnostic yield of robotic-assisted bronchoscopy (RB) may be inferior to that of transbronchial needle biopsy (TTNB). This notable discrepancy underscores the necessity for more comprehensive and systematically structured studies aimed at better defining the specific role of RB in the diagnostic evaluation of lung nodules. Such investigations could yield precise insights into the optimal scenarios for the application of this technique, including the types of lung nodules most amenable to successful diagnosis through RB [48-50].

Several critical factors can significantly influence the generally favorable diagnostic yield reported in robotic-assisted bronchoscopy. Among these are the prevalence of malignancy within the specific patient population being studied, which can impact the likelihood of obtaining a definitive diagnosis. Additionally, the selection criteria employed for participant inclusion in clinical studies can introduce variability and affect the perceived efficacy of RB. Furthermore, the issue of publication bias, where studies with positive results are more likely to be published, may skew the existing body of research, leading to an exaggerated perception of the success of RB in clinical practice [47-50].

For a comparative analysis of the efficacy of CT-guided transbronchial needle aspiration (CT-TTNA) versus newer modalities, relevant data are mentioned in Table **1**. This table provides a clear overview of the respective success rates, procedural complications, and patient outcomes associated with these diagnostic approaches, thereby facilitating a more informed discussion on the optimal strategies for lung nodule diagnosis.

### 2.7 Summarizing Pros and Cons of Conventional versus Newer Techniques

CT-guided transthoracic needle biopsy (TTNB) is a widely used method for diagnosing lung nodules, particularly those located near the chest wall or in deeper regions of the lung. It is often preferred due to its high diagnostic yield. However, TTNB carries a risk of complications, such as pneumothorax, especially when crossing lung fissures or in patients with emphysema, which may limit its use in certain cases. A more detailed overview of its advantages and limitations is presented in Table **2**.

Bronchoscopic techniques, including electromagnetic navigational bronchoscopy (ENB), radial probe endobronchial ultrasound (RP-EBUS), and robotic bronchoscopy (RB), offer an alternative approach, particularly for patients at higher risk of pneumothorax. These methods also allow for the simultaneous sampling of multiple lymph nodes via EBUS when evaluating suspected malignancies. RB, an emerging technology, enhances bronchoscopic access to peripheral lung nodules, addressing some limitations of traditional bronchoscopy.

**Fig (1):**
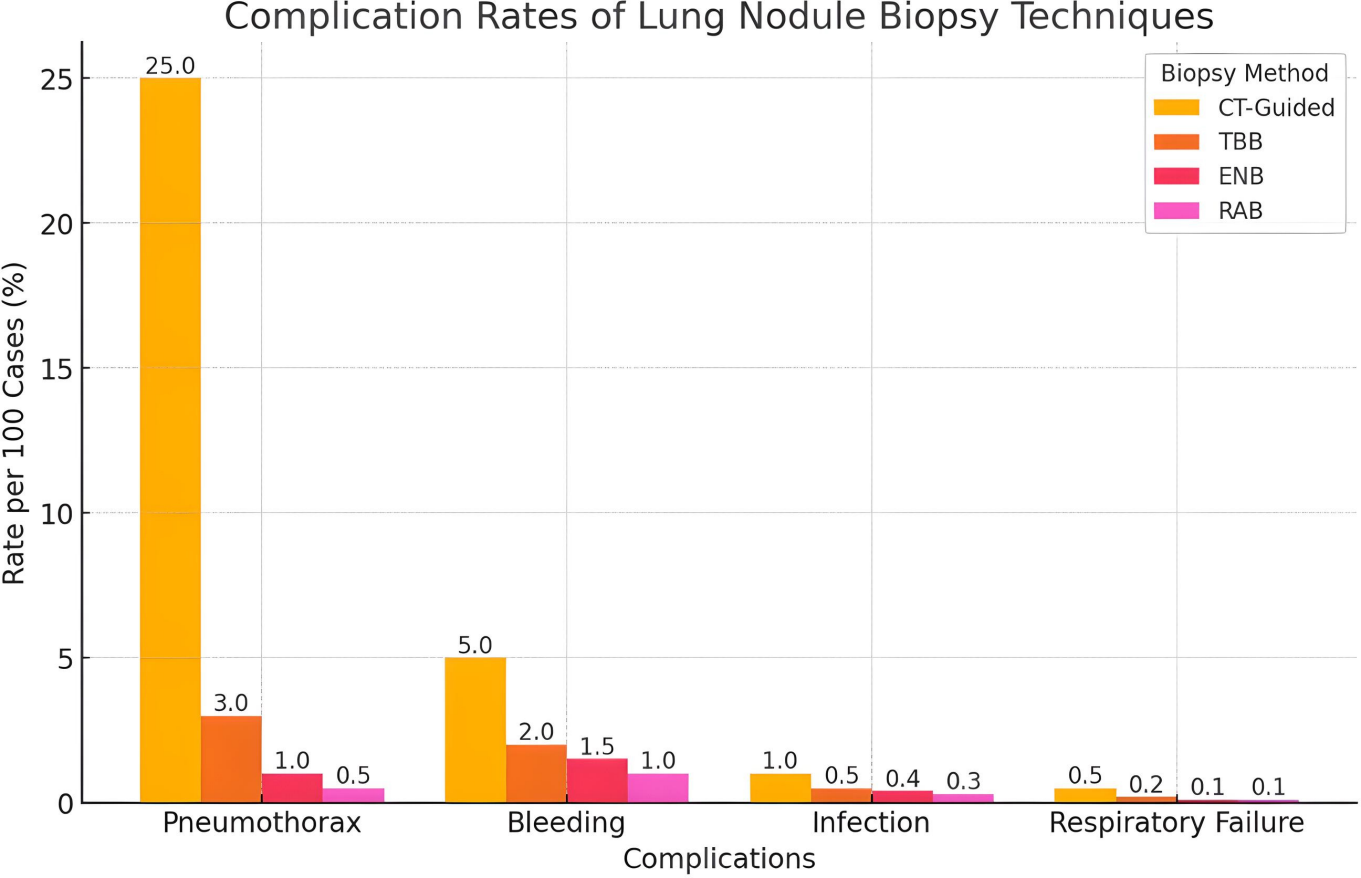
Complication Rates of Various Lung Nodule Biopsy Techniques.

While RB generally has a favorable safety profile (Fig. **1**), current research suggests that its diagnostic yield may be lower than that of TTNB. However, variability in study populations, malignancy prevalence, and potential publication bias can influence these findings. Complication rates for bronchoscopic approaches, including pneumothorax and bleeding, are generally low [50, 51].

Cost-effectiveness analyses suggest that ENB may be a viable alternative to TTNB, particularly when factoring in procedural risks, healthcare costs, and patient selection. While newer bronchoscopic techniques may have higher initial costs, they can be cost-effective if applied to appropriately selected patients. However, uncertainty remains regarding the extent to which the reduced complication rates and potential clinical benefits of ENB outweigh its higher procedural costs compared to TTNB in diagnosing malignant pulmonary nodules [50-54]. When suitable, RB may be considered as an option for lung nodule biopsy, with the choice of modality depending on patient characteristics, lesion location, and institutional expertise. A comparative analysis of these approaches is presented in Table **2**.

### 2.8 Ways to Reduce Technical Errors during CT-TTNA/TTNB and Bronchoscopic Procedures

Factors, such as a patient moving during a procedure and causing image blurriness, can impact the diagnostic accuracy of CT-TTNA or bronchoscopy. To mitigate this factor, various studies have suggested integrating error mitigation techniques, deep learning models, Artificial Intelligence (AI), and image deblurring methods to improve diagnostic accuracy. The AI-based segmentation in CT scans could help localize lung nodules better, thereby improving the success rate of biopsies or targeted bronchoscopic procedures. AI-based models trained on multimodal imaging (CT, PET, bronchoscopy data) could improve decision-making by predicting which biopsy method is more likely to succeed based on lesion location, size, and surrounding structures. AI models can assist pulmonologists and interventional radiologists in providing real-time procedural guidance to reduce human error in lesion detection and improve lesion localization, and recommend the optimal biopsy approach based on imaging and procedural data [55]. Similarly, MedDeblur helps to reduce motion artifacts and improve image clarity. It helps reduce complications and plan a better trajectory for the needle by improving real-time visualization of airway anatomy [55, 56]. Additionally, implementing better procedural protocols can lead to more accurate diagnoses and improved patient outcomes through advanced error techniques [56]. Fig. (**1**) shows the complication rates of various lung biopsy techniques.

This bar chart compares complication rates of different lung nodule biopsy methods, including CT-Guided Biopsy, Conventional Transbronchial Biopsy (TBB), Electromagnetic Navigational Bronchoscopy (ENB), and Robotic-Assisted Bronchoscopy (RAB). The y-axis represents the complication rate per 100 cases (%), while the x-axis lists different complications: pneumothorax, bleeding, infection, and respiratory failure.

Each bar represents the complication rate for a given technique, with numeric values displayed on top. CT-guided biopsy has the highest pneumothorax rate (25%), whereas RAB has the lowest across all categories. The rates are derived from literature sources and reflect the approximate percentage of cases experiencing each complication [57, 58].

### 2.9 Cost Comparison between Techniques

The cost of the procedure depends not only on the modality of lung nodule biopsy but also on location (inpatient versus outpatient), medical insurance coverage, post-procedural complications, and the need for a repeat biopsy, among others. Hence, there is a wide range of variability in determining the cost of various modalities of lung nodule biopsy Table **3**.

While the initial cost of using advanced technologies, such as robotic-assisted lung nodule biopsy, might be higher than traditional methods, the reduction in complication rates and the potential decrease in the need for repeat biopsies could lead to overall cost savings in the patient's diagnostic journey [59-61]. Fig. (**2**) shows the comparison of the transthoracic versus bronchoscopy approach for lung nodule biopsy based on patient and lesion characteristics.

### 2.10 Integration of Newer Technology

The optimization of biopsy techniques for lung nodules relies not only on procedural advancements but also on strategies to mitigate errors and enhance diagnostic accuracy. Recent developments in measurement error mitigation, such as those described in quantum computing research, have parallels in procedural optimization for guided biopsy techniques, where reducing inaccuracies in navigation and tissue sampling can significantly improve diagnostic yield [62]. Additionally, advancements in deep learning-based image analysis, as applied in brain tumor segmentation and ophthalmic disease analysis, underscore the role of multi-modality imaging and enhanced visualization in improving the precision of bronchoscopic navigation and biopsy [62, 63]. Furthermore, techniques, such as medical image deblurring and adaptive ensemble deep learning models, hold promise for refining imaging quality and diagnostic decision-making, which are critical for both bronchoscopic and CT-guided approaches [62]. Incorporating these emerging technologies into lung nodule evaluation may help bridge existing gaps in diagnostic accuracy, procedural safety, and real-time error correction, ultimately optimizing biopsy outcomes.

## CONCLUSION

Over the past few decades, CT-guided fine-needle aspiration and biopsy (TTNA/TTNB) have been recognized as the gold standard for diagnosing pulmonary lesions in the peripheral and mid-lung zones. This technique has provided reliable results and has been integral in guiding treatment decisions. However, recent advancements in imaging technologies and minimally invasive procedures have introduced new modalities that may offer improved accuracy and reduced complication rates.

These innovative approaches, which often integrate advanced imaging techniques, such as ultrasound, MRI, or hybrid methods, warrant re-evaluating our current practices. Nevertheless, comprehensive prospective studies must be conducted to accurately position these newer modalities as the gold standard. This research should focus on gathering relevant clinical data, comparing outcomes, and understanding the implications of adopting these newer methods in routine clinical practice. It would be beneficial to fully transition to these alternatives only when substantial evidence is collected, further validating their efficacy and safety in diagnosing pulmonary lesions.

## Figures and Tables

**Fig. (2):**
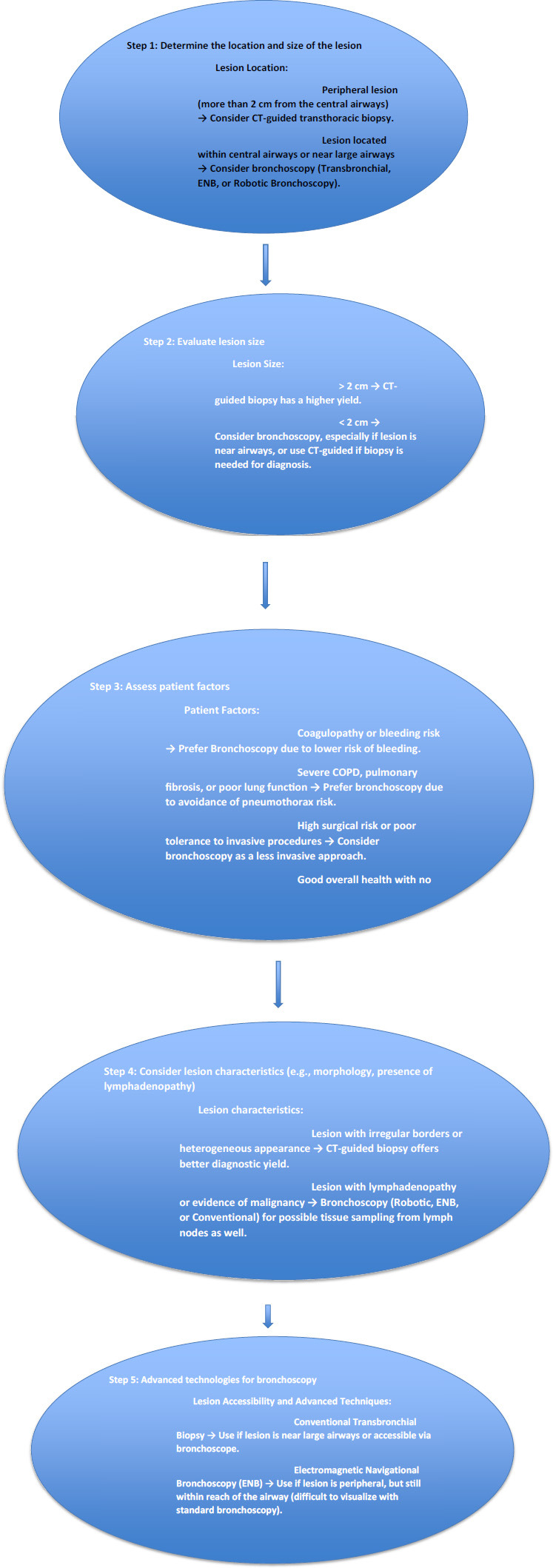
Flowchart showing when to use the transthoracic *versus* bronchoscopy approach for lung nodule biopsy based on patient and lesion characteristics.

**Table 1:** Comparing diagnostic yield and safety of CT-TTNA/TTNB with newer modalities.

**Procedure**	**Type of Study**	**Number of Cases /Lesion**	**Diagnostic** **Yield**	**Detection Rate of Genetic Markers**	**Rate of Pneumothorax**
CT-TTNA/TTNB Zhu *et al.* and Rickets *et al.* [37, 50]	Prospective	177 cases	97.7%	98.9%	28.8% (in 80 mm needle) 9.1% (in 10 mm needle)
CT-TTNA/TTNB (Tuna *et al.* and Rickets *et al.*) [14, 50]	Prospective	105 cases	78%-92%	NA	10%
EBUS-GS (Zhu *et al.* and Rickets *et al.*) [37, 50]	Prospective	150 cases	64.0%	96.1%	0.7%
Robotic bronchoscopy (Rickets *et al.* and Chaddha *et al.*) [50, 51]	Retrospective	167 lesions	69.1%-77%	NA	3.6%
Guided bronchoscopy, including VB, EMB, and RP-EBUS (Rickets *et al.* and Memoli *et al.*) [50, 52]	Meta-analysis	3052 lesions	70%	NA	1.5%
Navigation bronchoscopy, including CBCT, RB, tomosynthesis-guided EMN (Kops *et al.* and Rickets *et al.*) [46, 50]	Meta- analysis	10,682 lesions	77.5%	NA	2.5%

NA: Not Available

**Table 2:** Comparison between conventional (CT Guided TTNB) and newer image-guided bronchoscopic techniques.

Procedure	Advantages	Limitations
CT Guided TTNB	● Cost-effective (Birchard [6]. ● Preferred for the peripheral lesions in proximity to the chest wall ● Preferred for lesion size less than 2cm (Birchard *et al.* and Bardino *et al.*), [6, 8]. ● Ability to obtain tissue independent of the tumour-bronchus relationship ● Higher diagnostic accuracy for distinguishing between benign and malignant diseases, ranging from 77-94% (Birchard *et al.*, Bardino *et al.*, and Ohno *et al.*) [6, 8, 19]. ● Lesser duration of hospital stay due to minimal requirement of additional procedures, such as thoracotomies or bronchoscopies (Birchard *et al.*) [6]. ● Can be performed by general pulmonologists as well as interventional radiologists (Munoz *et al.*) [12].	● High rate of complications, such as pneumothorax, ranging from 19% to 25% (DiBardino *et al.* and Heerink *et al.*) [8, 9]. ● Chance of inadequate sampling and false negative results. ● Unable to assess the mediastinal lymph nodes during the procedure (Bardino *et al.*) [8]. ● Increased chances of needing additional biopsies (Munoz *et al.*) [12].
Image-guided bronchoscopy methods	● Low rate of complications, *i.e.*, 1.0% (95% CI 0.2-1.8%) (Asahina *et al.*) [38]. ● Improved visualization and access for small and hard-to-reach nodules (Zhang *et al.*) [45]. ● Appropriate in patients with high risk of pneumothorax (Eberhardt *et al.*) [48]. ● Despite the initial higher cost, in the long run, with additional navigational techniques and a reduced need for second-pass biopsies, it provides a more comprehensive approach to diagnosis (Gex *et al.* and Wang *et al.*) [47, 52].	● Lower overall diagnostic yield of 73.8% [95% confidence interval (CI) 70.9-76.8%] (Asahina *et al.*) [38]. ● Requires more institutional expertise (Kalchiem-Dekel *et al.*) [40]. ● Requires highly skilled physicians ● Diagnostic accuracy is lower for lesions <20 mm at 67.4% (95% CI 63.3-71.5%) (Nadig *et al.*) [49]. ● Initial higher cost (Gex *et al.* and Wang *et al.*) [47, 52].

**Table 3:** Cost comparison of lung nodule biopsy techniques based on the evidence available.

Lung nodule biopsy technique	Approximate cost per procedure
CT-Guided Biopsy /Percutaneous biopsy	$1028.52 average, depending on various other factors [59].
Electromagnetic Navigational Bronchoscopy (ENB)	ENB biopsy increases average costs by $3719 per case [60].
Robotic bronchoscopy	No studies available to accurately determine the cost of a robotic-assisted bronchoscopy [61]. Estimated to be associated with higher costs as compared to ENB.

## LIST OF ABBREVIATIONS

RB: = Robotic bronchoscopy
VBN: = Virtual Bronchoscopic Navigation
ENB: = Electromagnetic Navigation Bronchoscopy
RP-EBUS: = Radial Probe Endobronchial Ultrasound
CT: = Computed Tomography
NLST: = National Lung Screening Trial
EBUS: = Endobronchial ultrasound biopsy
TTNA: = Transthoracic Needle Aspiration
